# EP09 COVID-19 and rheumatic disease clinical characteristics and outcomes of rheumatic disease patients hospitalised with COVID-19: a single centre experience

**DOI:** 10.1093/rap/rkaa052.008

**Published:** 2020-11-03

**Authors:** Myrto Cheila, Ali Elrashid, Nicola Erb, Karen Douglas, Christos Koutsianas

**Affiliations:** Dudley NHS Foundation Trust, Dudley, United Kingdom

## Abstract

**Case report - Introduction:**

There is limited evidence on the outcomes of COVID-19 in patients with rheumatic diseases. Since severe COVID-19 is characterised by hyperinflammation, the effects of a pre-existing inflammatory state and immunomodulatory treatments on the disease’s course are of great interest. Even though patients in this group are perceived of being at higher risk for severe disease, most of the relevant observational studies have had controversial results.

We aimed to investigate the clinical characteristics and outcomes of patients with a history of rheumatic disease and COVID-19 related admission in our centre.

**Case report - Case description:**

We conducted a retrospective observational study of COVID-19 RT-PCR positive admissions of patients coded as having rheumatic disease in the Dudley Group NHS Foundation Trust between April 1st and June 24^th^, 2020. Data were extracted from the patient clinical notes and electronic medical records and were captured and analysed on MS Excel. Among 613 COVID-19 related admissions, 19 (3.1%) patients were coded as having comorbid rheumatic disease. 8 cases were excluded from further analysis as, upon records review, they had osteoarthritis or a history of gout. Thus, the true incidence of comorbid rheumatic disease among COVID-19 related hospitalisations was even lower at 11/613 (1.8%). The mean age of the patients was 80.5 ± 8.06 years, 9/11 (81.8%) were female and 8/11 (73%) were Caucasian. In the vast majority the diagnosis was that of Rheumatoid Arthritis (9/11, 81.8%), while one patient had Polymyalgia Rheumatica and another Systemic Lupus Erythematosus. Only a quarter of the patients had moderate (2, 18%) or high (1, 9%) disease activity on their most recent outpatient visit. In terms of antirheumatic medication, 5 (45%) patients were on regular Prednisolone (20% <5mg, 80% 5-10mg), 8 (72%) were on cs DMARDs and only 1 (9%) on bDMARDs. A substantial proportion of the patient cohort also suffered with other comorbidities. 8/11 (72.7%) patients had arterial hypertension, 5 (45.4%) had a history of cardiovascular disease, 3 (27.3%) obesity (BMI 30+), 3 (27.3%) previous history of cancer, 2 (18.2%) COPD, 2 (18.2%) CKD, 1 (9%) Interstitial lung disease, 1 (9%) Diabetes, 1 (9%) Cerebrovascular disease and 1 (9%) immunodeficiency. During their admission, 7 (63.6%) patients required supplemental oxygen therapy. Unfortunately, 5/11 (45.5%) patients had a fatal outcome.

**Case report - Discussion:**

In our cohort, the percentage of patients with rheumatic diseases among inpatients with confirmed COVID-19 infection was low (∼1.8%). Potential explanations for this observation could be a beneficial effect of concomitant antirheumatic treatment (steroids and/or DMARDs) in controlling hyperinflammation, but also rheumatic disease patients’ increased risk awareness and thus increased compliance with viral spread mitigation measures (‘shielding’) as per Government guidelines. We also note the low rate of biologic DMARD use with only 1 (9%) inpatient having received Rituximab for RA.

**Case report - Key learning points:**

The incidence of rheumatic disease among COVID-19 related admissions in our centre was exceptionally low. Per recently published reports from worldwide registries, older age and multiple comorbidities appear to drive the risk for hospitalisation, need for oxygen supplementation and fatal outcome. Better understanding of the effect of DMARDs on COVID-19 severity requires further investigation, perhaps with SARS-CoV-2 antibody studies, but our observations appear to be reassuring.

